# The Effects of Different Inoculant Agents on the Growth of *Cymbidium faberi* and the Characteristics of Soil Bacterial Communities

**DOI:** 10.3390/biology15110872

**Published:** 2026-05-31

**Authors:** Xue Mao, Li Liu, Yongyao Liu, Zhuxin Mao

**Affiliations:** 1 Zhen’an County Wildlife and Natural Forest Protection and Management Center, Shangluo 711500, China; xm602362140@163.com (X.M.); yl879681107@163.com (Y.L.); 2 Xi’an Botanical Garden of Shaanxi Province, Institute of Botany of Shaanxi Province, Xi’an 710061, China; 3 Shaanxi Engineering Research Centre for Conservation and Utilization of Botanical Resources, Xi’an 710061, China

**Keywords:** *Cymbidium faberi*, microbial agent, ecological network, bacterial community composition

## Abstract

This study explored the effects of one mineral fertilizer and two microbial inoculants and their combined applications on soil physicochemical properties, bacterial community structure, and plant growth of *Cymbidium faberi* under potted cultivation, aiming to provide theoretical and technical support for the sustainable cultivation of ornamental orchids. A single-factor randomized block experiment was designed with eight treatments: control (CK), mineral sulfosulfuric acid potassium (HF), Bacillus subtilis (KC), *Trichoderma harzianum* (HC), mineral sulfosulfuric acid potassium + Bacillus subtilis (HK), mineral sulfosulfuric acid potassium + *Trichoderma harzianum* (HH), Bacillus subtilis + *Trichoderma harzianum* (KH), and mineral sulfosulfuric acid potassium + Bacillus subtilis + *Trichoderma harzianum* (HKH). Plant growth traits, soil properties, and soil bacterial community characteristics were measured. The effects of inoculant agents on *Cymbidium faberi* growth, soil environment, and bacterial community, as well as their interaction relationships, were systematically analyzed. The combination of three inoculants significantly promoted plant height and leaf thickness in *Cymbidium faberi*. Compared with CK, the relative abundance of *Pseudomonadota* and *Bacteroidota* in HH treatment increased by 6.0% and 11.0%, respectively, while the relative abundance of *Acidobacteriota* and *Verrucomicrobiota* decreased by 6.0% and 12.0%, respectively. Venn diagram analysis revealed 146 ASVs shared among all treatments. KC, HC, and HF had more unique ASVs, whereas HK and HKH had the fewest. Principal component analysis (PCA) was used to visualize differences in bacterial community structure. Significant differences among treatments were confirmed using ANOSIM. Ecological network analysis indicated predominantly positive (cooperative) associations among bacterial taxa, with HKH showing the highest proportion of positive edges, suggesting stronger bacterial cooperation. Correlation analysis showed that *Patesibacteria*, *Acidobacterita*, and *Planctomycetota* were significantly negatively correlated with pH and TP, while *Bacteroidota*, *Actinomycetota*, and *Methylomirabilota* were significantly positively correlated with pH. The Mantel analysis revealed a significant positive correlation between bacterial community composition and richness and pH. Further analysis using the structural equation model revealed that soil nutrients and bacterial communities were the main factors affecting plant growth. This study clarifies the response rules of plant growth, soil physicochemical properties and rhizosphere bacterial communities to different mineral fertilizer and microbial inoculant combinations, and provides a practical basis for the rational screening of functional inoculants and the construction of healthy rhizosphere microecosystems in *Cymbidium faberi* pot cultivation.

## Simple Summary

Researchers have always hoped to find simple and effective methods to cultivate potted *Cymbidium faberi*. This study selected microbial agents and conducted experiments with different combinations to observe the effects of various treatments on the growth of *Cymbidium faberi* and the condition of the soil. The test results showed that the combination of fertilizer and two types of microorganisms could significantly promote the growth of *Cymbidium faberi* and also change the survival state of microorganisms in the soil. The research confirmed that soil microbial agents directly affect the growth condition of *Cymbidium faberi*. A reasonable combination of microbial agents can create a more suitable soil environment for the growth of *Cymbidium faberi*. The results of this research can help anyone cultivate healthy *Cymbidium faberi* and also promote the healthy development of the ornamental flower cultivation industry.

## 1. Introduction

*Cymbidium faberi* is a perennial herbaceous orchid with high ornamental, cultural, and economic value. It is one of the famous traditional flowers in China and holds an important position in the flower cultivation industry [1]. However, traditional orchid cultivation relies heavily on chemical fertilizers and pesticides to maintain growth and prevent diseases, and excessive use over time can cause soil compaction, salinization, nutrient imbalance, and severe damage to soil microbial diversity, structure, and function [2]. Such damage often leads to the decline of beneficial microorganisms and the accumulation of pathogenic bacteria, which in turn further inhibits the improvement of the quality and yield of *Cymbidium*, and also limits the sustainable development of the orchid flower industry, forming a vicious cycle [3]. Although green cultivation technologies have been gradually explored in ornamental plants, targeted regulation strategies for *Cymbidium faberi* cultivation and soil microecological improvement remain insufficient. In this context, the need to seek environmentally friendly and sustainable cultivation auxiliary strategies is urgent. Microbial inoculants and humic-based amendments, as live products containing specific and highly active beneficial microorganisms (such as mineral-source sulfosulfuric acid potassium, Bacillus subtilis, Trichoderma, etc.), are regarded as a promising green biological solution due to their multiple potential effects such as improving soil, promoting growth, enhancing resistance, and inhibiting diseases [4]. Microorganisms participate in the soil nutrient cycle, maintain soil structure, and regulate crop growth [5]. The application of microbial inoculants and mineral-derived potassium sulfo-fulvate acts—either directly or indirectly—to decompose soil organic matter and synthesize the nutrients required for plant growth. Concurrently, the metabolic activities of these microorganisms improve soil aggregate structure, while interactions among microbial populations inhibit the proliferation of harmful bacteria [6,7]. The application of beneficial microorganisms in flower cultivation can effectively improve soil microbial abundance, optimize the rhizosphere microenvironment, and promote plant growth. Meanwhile, it helps reduce soil-borne disease incidence and chemical pesticide reliance, thereby further improving flower quality [8]. Studies have shown that the addition of microbial inoculants can promote plant growth, enhance plant resistance, and increase yield and quality [9]. Some studies have also shown that the addition of microbial inoculants increases the available nutrient content in the soil, increasing the proportion of bacteria and actinomycetes in the microorganisms [10]. Nevertheless, most relevant studies focus on food crops and garden plants, and the synergistic effects and mechanisms of different microbial inoculants on *Cymbidium faberi* are still poorly understood. Moreover, microbial inoculants can not only improve soil structure and soil fertility, but also enhance the disease resistance and stress tolerance of plants [11]. Currently, in flower cultivation, many types of soil conditioners and growth promoters are used, among which mineral sources such as sulfuric acid, potassium, Bacillus subtilis, and Trichoderma are three widely used and well-characterized inoculants. Mineral-source sulfosulfuric acid potassium, as a derivative of humic acid, has the functions of improving soil structure, activating soil nutrients, and promoting the root development and nutrient absorption of plants [11]. Bacillus subtilis, as a beneficial spore-forming bacterium, can inhibit the reproduction of harmful microorganisms in the soil by producing antibacterial substances, regulate the balance of soil microbial communities, and secrete growth-promoting substances to enhance plant stress resistance [12]. Trichoderma can not only inhibit soil-borne pathogens but also form a symbiotic relationship with plants, promoting plant growth and improving soil properties [13].

At present, there are many reports on the application of various microbial inoculants on a single crop, but there are relatively few specialized studies on the cultivation of *Cymbidium*, and most studies focus on the application effect of a single inoculant, with less research on the combined application of different functional inoculants. There is no clear understanding of the comprehensive regulatory effects of the single and combined applications of mineral-source sulfosulfuric acid potassium, Bacillus subtilis, and Trichoderma on the growth and development of *Cymbidium*, nor is there a systematic exploration of the coordinated changes in soil properties and soil microbial community structure after inoculant application. At the same time, the application effect of microbial agents is greatly influenced by factors such as application dosage, ratio method, and cultivation objects. Currently, there is no unified standard for the appropriate dosage and optimal ratio of such microbial agents in *Cymbidium faberi* cultivation, which leads to problems such as unreasonable dosage, chaotic ratio, and unstable application effects in actual application, seriously restricting the promotion and application of microbial agents in *Cymbidium faberi* green cultivation. Based on the current development demand of *Cymbidium faberi* cultivation and existing research gaps, five-year-old healthy *Cymbidium faberi* seedlings with consistent growth performance were selected as experimental materials. A total of eight treatments were established in this study, including three single-agent applications, three pairwise combined applications, one three-agent mixed application, and one blank control without any inoculant addition. Through pot experiments, this study systematically investigated the effects of treatments involving varying ratios of soil inoculants on the growth characteristics of *Cymbidium* orchids and the structure of the soil microbial community, thereby identifying the efficacy of different amendments and determining the optimal ratio scheme. We hypothesized that (1) combined inoculant treatments would promote C. faberi growth more than single inoculant treatments by increasing beneficial rhizosphere bacteria, and (2) inoculant treatments would reduce bacterial network complexity while increasing network stability. Our research on the changes in bacterial communities caused by different treatment methods is helpful for formulating strategies to manage the composition of soil microbial communities, thereby achieving the goal of promoting high-yield and high-quality plants.

## 2. Materials and Methods

### 2.1. Test Material

Test plants: *Cymbidium faberi*.

Test soil: The pot experiment was conducted using soil collected from Zhenghe Village, Gaofeng Town, Zhen’an County, Shangluo City, Shaanxi Province. The soil is a yellow–brown sandy loam. Test plants were five years old *Cymbidium faberi*; initial plant height and soil physicochemical properties (pH, SOC, TN, TP) are shown in Table 1.

Three inoculant agents were tested: For mineral-derived potassium sulfohumate, the manufacturer-recommended application rate is 60 kg/ha^2^; in this experiment, the applied rate was 120 kg/ha^2^ (approximately 12 g/m^2^). The flowerpots selected for this experiment have a diameter of 32 cm, yielding an individual surface area of 0.07 m^2^; thus, the required dosage per pot is approximately 12 × 0.07 = 0.84 g, or 0.84 g × 12 ≈ 9.68 g (rounded to 10 g). For *Bacillus subtilis*, the manufacturer-recommended rate is 15–30 kg/ha^2^, whereas the rate applied in this experiment was 60 kg/ha^2^ (6 g/m^2^). For *Trichoderma harzianum*, the manufacturer-recommended rate is 15–30 kg/ha^2^, whereas the rate applied in this experiment was 60 kg/ha^2^ (6 g/m^2^).

### 2.2. Test Method

The test was conducted from July to October in 2025. A single-factor random block design was adopted, with a total of 8 treatments. CK: control group, no microbial fertilizer was added; HF: 12 g/m^2^ mineral sulfosulfuric acid potassium was added; KC: 12 g/m^2^ Bacillus subtilis was added; HC: 12g/m^2^ *Trichoderma harzianum* was added; HK: 6 g/m^2^ mineral sulfosulfuric acid potassium + 6g/m^2^ Bacillus subtilis; HH: 6 g/m^2^ mineral sulfosulfuric acid potassium + 6g/m^2^ *Trichoderma harzianum*; KH: 6 g/m^2^ Bacillus subtilis + 6g/m^2^ *Trichoderma harzianum*; HKH: 6g/m^2^ mineral sulfosulfuric acid potassium + 6g/m^2^ Bacillus subtilis + 6g/m^2^ *Trichoderma harzianum*. Each treatment had 4 replicates, totaling 32 *Cymbidium faberi* plants. The experiment selected five healthy and uniformly growing *Cymbidium faberi* as the test subjects. The planting method of *Cymbidium faberi* was potted, with a flowerpot diameter of 32 cm and a flowerpot depth of 25 cm. The three types of amendments were individually dissolved in water—all exhibiting excellent water solubility—in accordance with the quantities specified in the experimental design. They were then added to the flowerpots in the prescribed amounts. To ensure uniform concentration, the volume of the amendment solution added was standardized at 1 L per pot; furthermore, the solution was applied in three separate increments to prevent any leakage.

### 2.3. Sample Collection and Measurement

The height of *Cymbidium faberi* plants was measured using a ruler, and the thickness of *Cymbidium faberi* leaves was measured using a vernier caliper. At the same time, the relative content of chlorophyll in the leaves was measured using a SPAD-502 Plus chlorophyll analyzer [14]. Soil samples from 0–20 cm of the rhizosphere were collected. The soil samples were divided into two parts for preservation: one part of the soil samples was naturally air-dried for the determination of soil factor indicators (pH value, total nitrogen, total phosphorus, organic matter); the other part of the soil samples was quickly immersed in liquid nitrogen and then stored in a −80 °C refrigerator for microbial community analysis.

pH was measured using a pH meter (the water-to-soil ratio was 2.5:1) [15]; organic matter content was determined using the potassium dichromate titration method [16]; total nitrogen content was determined using a fully automatic Kjeldahl nitrogen analyzer [17]; total phosphorus content was determined using the HClO_4_-H_2_SO_4_ method [18] (Table 2).

### 2.4. Soil DNA Extraction, High-Throughput Sequencing, and Bioinformatics Analysis

DNA was extracted from soil samples using a FastDNA^®^ SPIN Kit for Soil (MP Biomedicals, Agoura Hills, CA, USA). For each sample, approximately 0.5 g of fresh soil was weighed, and the extraction was performed following the kit’s instructions. The obtained total soil DNA was assessed for integrity via agarose gel electrophoresis, and DNA concentration and purity were measured using a UV-Vis spectrophotometer (NanoDrop 2000, Thermo Scientific, Waltham, MA, USA). Samples with clear and non-dispersed DNA bands and high purity and concentration were selected for subsequent amplicon sequencing. PCR amplification of the bacterial 16S rRNA gene was conducted using primers 515F (5′-GTGCCAGCMGCCGCGG-3′) and 907R (5′-CCGTCAATTCMTTTRAGTTT-3′) to target the V4–V5 region. PCR products were quantified, homogenized, filtered, assembled, and screened to remove chimeras using QIIME v1.9.1 software and the UPARSE pipeline. (v7.0.1090) Effective sequences with >97% similarity were clustered into ASVs using QIIME and the UCLUST (USEARCH v7.0.1090) sequence alignment tool, and the most abundant sequence in each ASV was selected as its representative sequence. Taxonomic information for each ASV was obtained by comparing representative sequences to the Silva 138 database. An ASV abundance matrix (ASV table) was constructed based on the number of sequences in each ASV for each sample. Community richness (Chao1 and ACE indices) and community evenness (Shannon and Simpson indices) were calculated. High-throughput sequencing was performed using the platform of Shanghai Meiji Biomedical Technology Co., Ltd. (Shanghai, China).

### 2.5. Data Processing

The bacterial community diversity and structural composition were analyzed using the MajorBio cloud platform (https://www.majorbio.com/ (accessed on 2 January 2026). Excel 2016 was used for data statistical analysis of soil properties and plant physiological conditions. Prior to one-way ANOVA, data normality was verified by the Shapiro–Wilk test, and variance homogeneity was examined using Bartlett’s test and Levene’s test to meet the statistical assumptions of ANOVA. One-way ANOVA was performed using SPSS 25.0, and significance difference analysis was conducted using multiple comparisons (LSD method, *p* = 0.05). Principal component analysis (PCA) was used to explore patterns and variation in the soil bacterial community structure among treatments based on ASV abundance data. Since PCA is an exploratory ordination technique, it was not used for statistical significance testing. To assess statistical differences in bacterial community composition among treatments, analysis of similarity (ANOSIM) was performed using Bray–Curtis dissimilarities with the vegan package in R (version 4.0.3). ANOSIM provides a nonparametric assessment of whether bacterial community composition differs significantly among treatment groups. Molecular ecological networks were constructed based on Spearman correlation analysis using R language. To ensure network reliability, only correlation pairs with a correlation coefficient |r| > 0.6 and a significance level of *p* < 0.05 were retained as valid network edges. The constructed networks were visualized with Gephi v0.9.2 software. Based on the topological characteristics of the nodes, the node attributes can be classified into 4 types: Module hubs (central nodes within a module with high connectivity, Zi > 2.5 and Pi < 0.62), Connectors (nodes connecting two modules with high connectivity, Zi < 2.5 and Pi > 0.62), Network hubs (central nodes in the entire network with high connectivity, Zi > 2.5 and Pi > 0.62), and Peripherals (peripheral nodes without high connectivity within or between modules, Zi < 2.5 and Pi < 0.62). Generally, the remaining 3 types of nodes (excluding Peripherals) are classified as key nodes. All bar charts and stacked column charts involved in this study were plotted using Origin 2021, and all figures were uniformly optimized and typeset with Adobe Illustrator 2020 for standardized presentation. Partial least squares path analysis is a structural equation model based on correlation, a method suitable for exploratory research, applicable to small sample sizes as well as large datasets, and which does not require multivariate normality assumptions [19]. In this study, PLS-PM was used to explore the influence path of microbial agents on soil bacteria, and the analysis was conducted using the plspm package in R language (Table 3).

## 3. Results and Analysis

### 3.1. Effects of Different Microbial Agents on the Growth Traits of Cymbidium faberi

**Figure 1:**
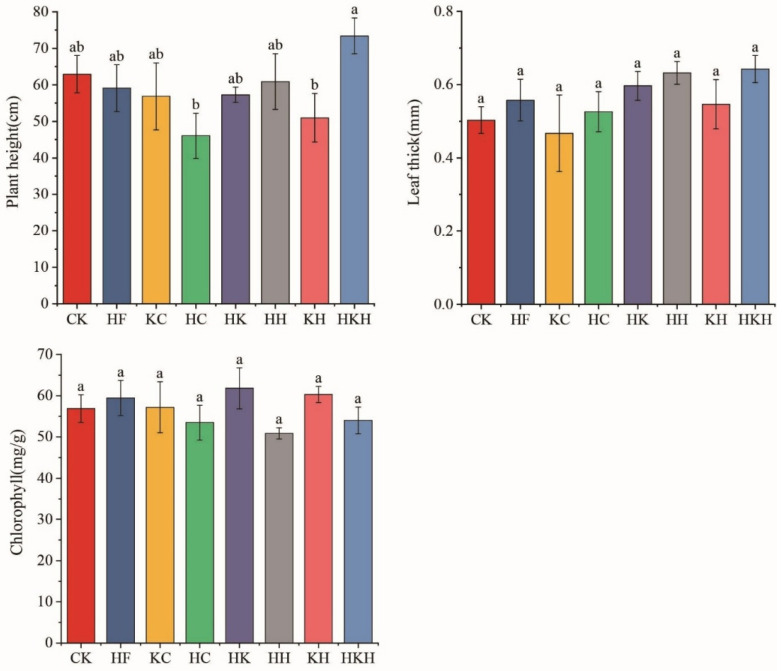
Growth and physiological indices of *Cymbidium faberi* under different treatments in a potted experiment conducted from July to October 2025. Note: CK: control group, no addition of any microbial agents; HF: added mineral sulfosulfuric acid potassium; KC: added Bacillus subtilis; HC: added *Trichoderma harzianum*; HK: added mineral sulfosulfuric acid potassium and Bacillus subtilis; HH: added mineral sulfosulfuric acid potassium and *Trichoderma harzianum*; KH: added Bacillus subtilis and *Trichoderma harzianum*; HKH: added mineral sulfosulfuric acid potassium, Bacillus subtilis, and *Trichoderma harzianum*. Different lowercase letters indicate significant differences after LSD test adjustment among each treatment (*p* < 0.05), and these values are the mean ± SE. The same as below.

This study measured various growth and physiological parameters—including plant height, leaf thickness, and chlorophyll content—under different treatment conditions (Figure 1). Compared with CK, the HKH treatment significantly enhanced both plant height and leaf thickness (*p* < 0.05), demonstrating the most optimal overall growth performance. In contrast, the HC treatment resulted in plant height and chlorophyll content that were significantly lower than those of the other treatments, indicating marked growth inhibition (*p* < 0.05). The HH treatment effectively increased leaf thickness, yet significantly reduced chlorophyll content. The KC treatment significantly decreased leaf thickness but exerted no significant effect on plant height or chlorophyll content. Finally, treatments such as HF, HK, and KH showed no significant differences compared to the control group, with only minor fluctuations observed in a few individual parameters.

### 3.2. Effects of Different Microbial Agents on Soil Properties

**Figure 2:**
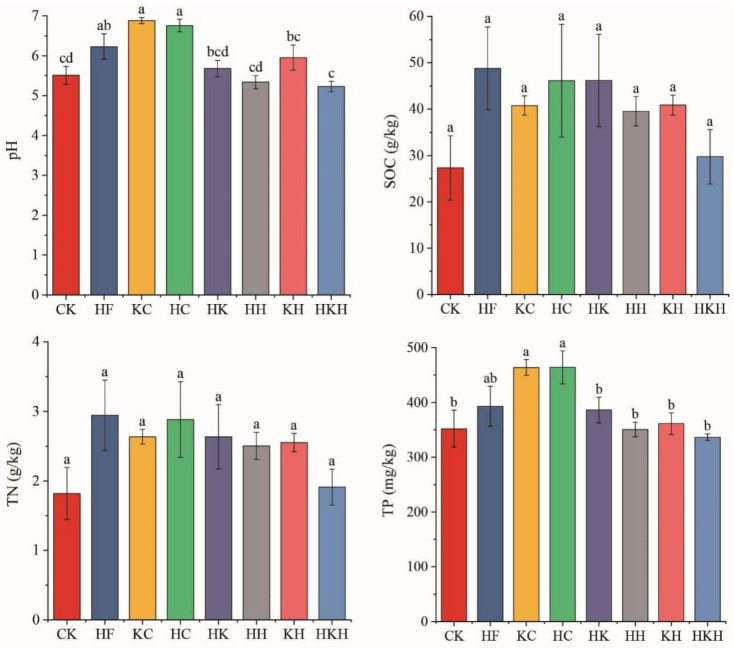
Changes in soil nutrients (SOC, TN, TP, pH) under different treatments in a potted *Cymbidium faberi* experiment conducted from July to October 2025. Different lowercase letters indicate significant differences after LSD test adjustment among each treatment (*p* < 0.05), and these values are the mean ± SE. The same as below.

As shown in Figure 2, compared with CK, the KC and HC treatments significantly increased soil pH and TP content (*p* < 0.05), while having no significant effect on SOC and TN. Compared with CK, the pH of the KC and HC treatments increased by 19.95% and 18.50%, respectively; the SOC of the HF and HC treatments increased by 43.40% and 40.80%, respectively; the TN of the HF and HC treatments increased by 38.27% and 36.90%, respectively; and the TP of the KC and HC treatments increased by 24.10% and 24.13%, respectively. Compared with CK, the HKH treatment decreased pH and TP by 5.08% and 4.40%, respectively.

**Figure 3:**
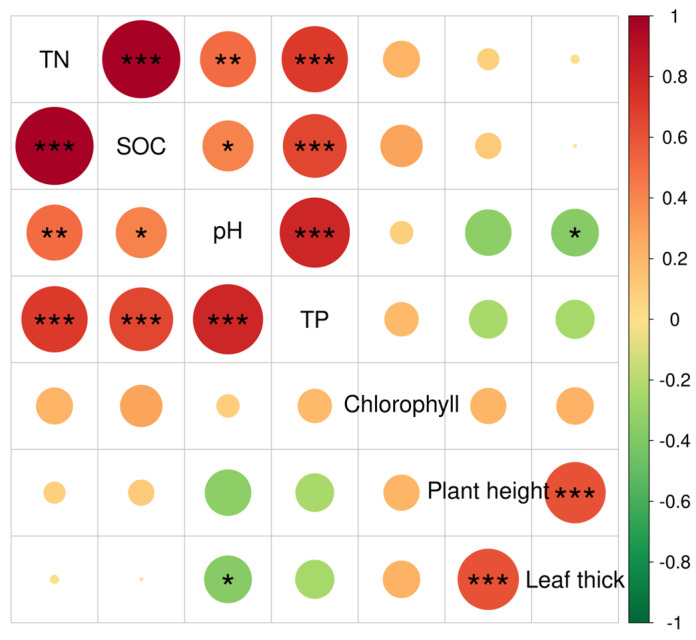
Correlation matrix among plant growth traits and soil nutrients (SOC, TN, TP, pH) under different treatments in a *Cymbidium faberi* experiment conducted from July to October 2025. The * symbol indicates the significance marking corresponding to the *p*-value. If the *p*-value is less than 0.001, it is represented by ***; if less than 0.01, by **; if less than 0.05, by *. Other values do not display the significance marking. SOC—soil organic carbon, TN—soil total nitrogen, TP—soil total phosphorus.

Further, through correlation analysis (Figure 3), pH was significantly positively correlated with SOC, TN and TP, and negatively correlated with leaf thickness (*p* < 0.05). TN was significantly positively correlated with SOC and TP, and TP was significantly positively correlated with SOC (*p* < 0.05). Stem height was significantly positively correlated with leaf thickness (*p* < 0.05).

### 3.3. Effects of Different Microbial Agents on Soil Bacterial Communities

**Figure 4:**
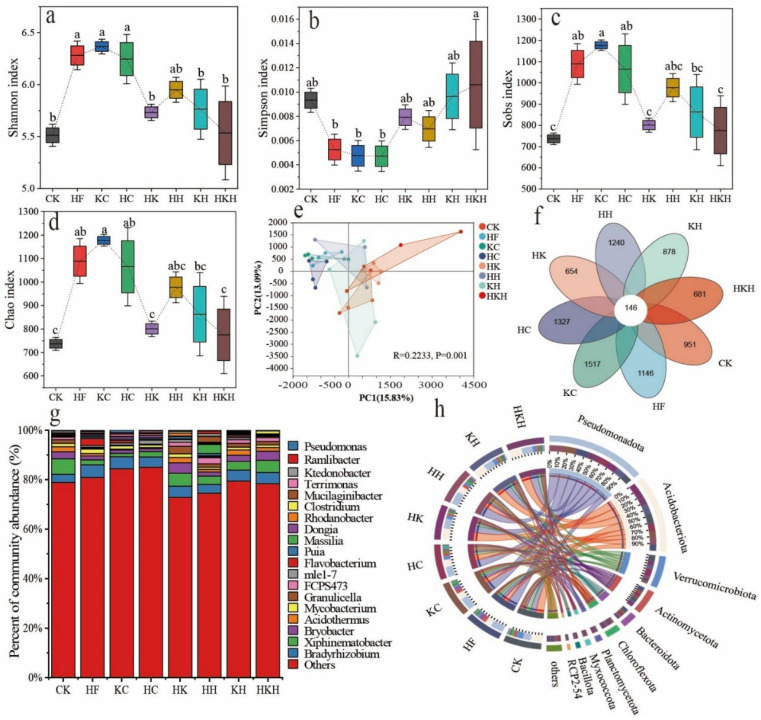
Composition of bacterial communities in *Cymbidium faberi* experiment conducted from July to October 2025. Note: (**a**) Represents the Shannon index of bacteria; (**b**) Represents the Simpson index of bacteria; (**c**) Represents the Sobs index of bacteria; (**d**) Represents the Chao1 index of bacteria different lowercase letters indicate significant differences among treatments (*p* < 0.05); (**e**) represents the principal coordinate analysis (PCA) of soil bacterial communities based on Bray–Curtis distance; (**f**) Venn diagram showing the number of shared and unique ASVs of bacteria among different treatments; (**g**) accumulative plots of soil bacteria at the genus level under different treatments; (**h**) network diagrams of soil bacterial communities at the phylum level under different treatments.

By conducting high-throughput sequencing of bacteria, the results were averaged based on the minimum sample size. After processing, a total of 12,414 ASVs were obtained, belonging to 35 phyla, 102 classes, 230 orders, 374 families, and 717 genera (Figure 4). At the phylum classification level, the top 10 relative abundances of bacterial communities in different treatments were *Pseudomonadota*, *Acidobacteriota*, *Verrucomicrobiota*, *Actinomycetota*, *Bacteroidota*, *Chloroflexota*, *Planctomycetota*, *Myxococcota*, *Bacillota*, and *RCP2-54*, accounting for 96.8% of total sequences and representing the dominant bacterial phyla. Compared with CK, the application of microbial agents increased the relative abundances of *Pseudomonadota* and *Bacteroidota*, with HH treatment showing a significant increase of 6.0% and 11.0%, respectively. The application of microbial agents reduced the relative abundances of *Acidobacteriota* and *Verrucomicrobiota*, with HH treatment showing significant decreases of 6.0% and 12.0%, respectively (Figure 4h). At the genus classification level, the top seven relative abundances of bacterial communities in different treatments were *Bradyrhizobium*, *Xiphinematobacter*, *Bryobacter*, *Acidothermus*, *Mycobacterium*, *Granulicella*, and *FCPS473*, and the relative abundances of these bacteria reached over 1.0% in each treatment, accounting for 20.73% of the total abundance, and 79.37% of the unclassified species. This indicates that there are still a large number of bacterial resources in the *Cymbidium faberi* soil that need to be further explored and studied under different treatments. Compared with CK, the application of microbial agents increased the relative abundance of *Bradyrhizobium*, with HF treatment showing a significant increase of 33.79%. The application of microbial agents decreased the relative abundances of Xiphinematobacter and *Bryobacter*, with KC treatment showing a significant decrease of 77.22% and 51.57%, respectively (Figure 4g).

The Venn diagram analysis revealed (Figure 4f) that there were 146 common ASVs among all treatments. The unique ASVs specific to CK, HF, KC, HC, HK, HH, KH, and HKH treatments were 951, 1146, 1517, 1327, 654, 1240, 878, and 681, respectively. The unique ASVs in the KC, HC, and HF treatments were more numerous, while the unique ASVs in the HK treatment were the least. Principal component analysis revealed (PCA) (Figure 4e) that there were significant differences in the microbial community structures among different treatments (*p* < 0.05), with the HKH treatment having the greatest difference from other treatments, while the HF, KC, and HC treatments clustered together, indicating that their microbial community structures were relatively similar. The diversity index and richness index of the bacterial community were revealed by an analysis (Figure 4a,b). Among them, the KC and HC treatments significantly increased the Shannon diversity index, while the HF and HC treatments significantly enhanced the Sobs and Chao richness indices (*p* < 0.05). The three treatments had a relatively large number of unique ASVs, and the community structures were similar. However, the HKH treatment significantly reduced the microbial diversity and richness, and the community structure differed the most from the other treatments (*p* < 0.05).

**Figure 5:**
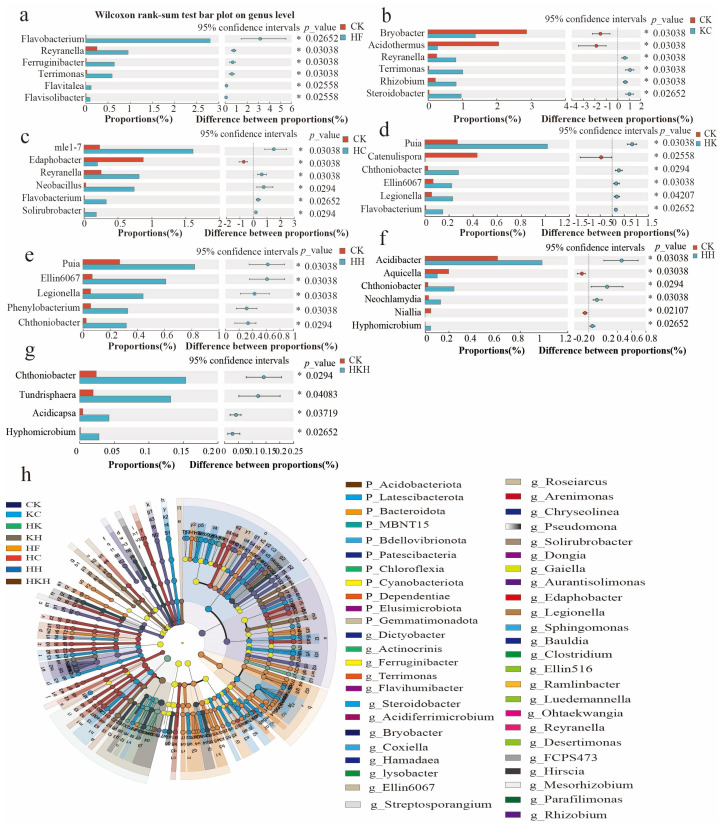
Analysis of differences in soil bacterial community composition among different treatments in a *Cymbidium faberi* experiment conducted from July to October 2025. Note: panels (**a**–**g**) show genus-level comparative bar charts based on the Kruskal–Wallis test with adjusted post hoc multiple comparisons, presenting significantly differential bacterial genera and their 95% confidence intervals (*p* < 0.05); asterisk * indicates *p* < 0.05. Linear discriminant analysis effect size (LEfSe) at the genus level was used to identify taxa with significantly differential abundance across treatments, which are shown as colored dots. (**a**) CK vs HF; (**b**) CK vs KC; (**c**) CK vs HC; (**d**) CK vs HK; (**e**) CK vs HH; (**f**) CK vs HH; (**g**) CK vs HKH. (**h**) Circos plot illustrating the taxonomic distribution of bacterial genera across all treatments, showing the relationship between phylum-level classification and genus-level abundance in different groups.

The differences in the soil bacterial communities (at the genus level) were determined through *t*-tests, and CK was used as the control (Figure 5a–g). Compared with CK, the HF treatment significantly increased the genera *Flavobacterium*, *Reyranella*, and *Ferruginibacter*; the KC treatment significantly increased the genera *Reyranella*, *Terrimonas*, and *Steroidobacter*; the HC treatment significantly increased the genera *mle1-7*, *Reyranella*, and *Neobacilla*; the HK treatment significantly increased the genera *Puia*, *Catenulispora*, and *Chthoniobacter*; the HK treatment significantly increased the genera *Puia*, *Catenulispora*, and *Chthoniobacter*; the HH treatment significantly increased the genera *Puia*, *Ellin6067*, and *Legionella*; the KH treatment significantly increased the genera *Acidibacter*, *Aquicella*, and *Chthoniobacter*; and the HKH treatment significantly increased the genera *Chthoniobacter*, *Tundrisphaera*, and *Acidicapsa*. The LEfSe analysis showed (Figure 5h) that at the phylum level, *Acidobacteriota*, *Latescibacterota*, *Bacteroidota*, and MBNT15 were the dominant groups, and there were significant differences in the distribution of these groups among the treatments (*p* < 0.05). At the genus level, the genera *Dictyobacter*, *Actinocrinis*, *Ferruginibacter*, and *Terrimonas* showed significant differences in different treatments. The KC and HC treatments significantly increased the genera *Bryobacter* and *Reyranella*, while the HK and HH treatments significantly increased the genera *Puia* and *Ellin6067* (*p* < 0.05).

**Figure 6:**
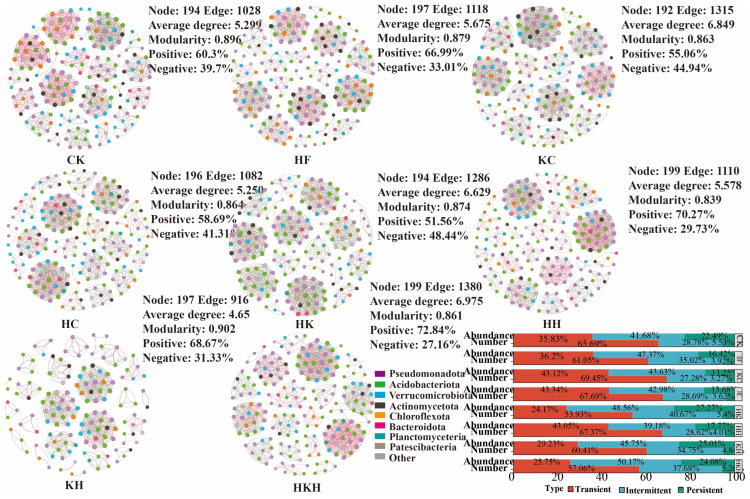
Analysis of the co-occurrence network of soil bacterial communities in *Cymbidium faberi* experiment conducted from July to October 2025. Note: the size of the nodes represents the degree (or the number of connected nodes). The red connecting lines indicate positive connections, while the green connecting lines indicate negative connections. The nodes are colored according to different classifications.

The addition of different microbial agents significantly alters the characteristics of the soil bacterial co-occurrence network (Figure 6). The analysis of the soil bacterial network revealed significant differences in the number of nodes, edges, average degree, and modularity among the various treatments. Among them, the HKH treatment had the highest number of nodes (199), edges (1380), and average weighted degree (6.975), indicating that the complexity of the soil bacterial co-line network in this treatment was higher, and the interactions between different species were also more complex. In contrast, the HC treatment had a lower number of nodes (196), edges (1082), and average degree (5.250), suggesting that the complexity of the soil bacterial co-line network in this treatment was lower, and the interactions between different species were simpler. The proportion of positive correlation edges was higher in the HH (70.27%) and HKH (72.84%) treatments, indicating stronger synergistic effects between microorganisms, while the proportion of negative correlation edges was higher in the KC (44.94%) and HK (48.44%) treatments, reflecting stronger competitive relationships. A comparative analysis of bacterial sensitivity to environmental conditions revealed that the higher the proportion of transient species, the lower the proportion of persistent species; this indicates that this category of bacteria exhibits a heightened sensitivity to environmental factors within the studied samples.

**Figure 7:**
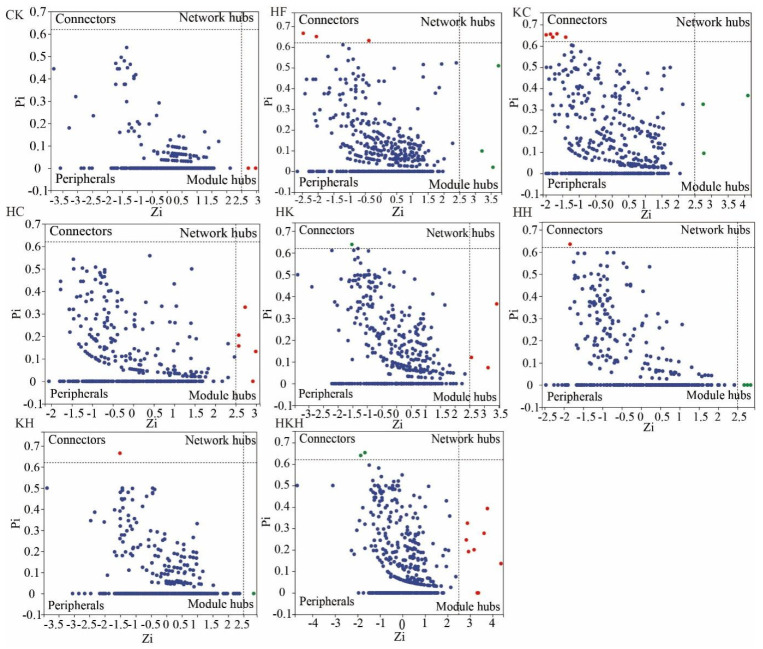
Analysis of intra-module connectivity (Zi) and inter-module connectivity (Pi) of soil bacteria modules in a *Cymbidium faberi* experiment conducted from July to October 2025. Based on the topological characteristics of the nodes, the node attributes can be classified into 4 types: Module hubs (central nodes within a module with high connectivity, Zi > 2.5 and Pi < 0.62), Connectors (nodes connecting two modules with high connectivity, Zi < 2.5 and Pi > 0.62), Network hubs (central nodes in the entire network with high connectivity, Zi > 2.5 and Pi > 0.62), and Peripherals (peripheral nodes without high connectivity within or between modules, Zi < 2.5 and Pi < 0.62). Generally, the remaining 3 types of nodes (excluding Peripherals) are classified as key nodes.

Further, through the analysis of the nodes in the soil bacterial co-occurrence network under different treatments (CK, HF, KC, HC, HK, HH, KH, HKH) (Figure 7), it was found that in the KC, HF, HK, HH, and HKH treatments, the number of Connectors and Module hubs was relatively higher, indicating that the bacterial networks under these treatments had stronger inter-module connections and core regulatory capabilities within the modules. The community interaction structure was more stable and complex. In the CK, HC, and KH treatments, most nodes were concentrated in the Peripherals area, and the number of Connectors and Module hubs was relatively lower, suggesting that the inter-module connections and core regulatory functions within the modules of the microbial networks in these treatments were relatively weak, and the community interaction structure was more loose. Additionally, in all treatments, the number of Network hubs (nodes with high Zi and high Pi) was extremely small, and only sporadically appeared in a few treatments, indicating that the bacterial networks under each treatment lacked absolute core regulatory nodes and relied more on Connectors and Module hubs to maintain the network structure.

### 3.4. Relationships Between Soil Bacterial Communities and Physicochemical Factors

**Figure 8:**
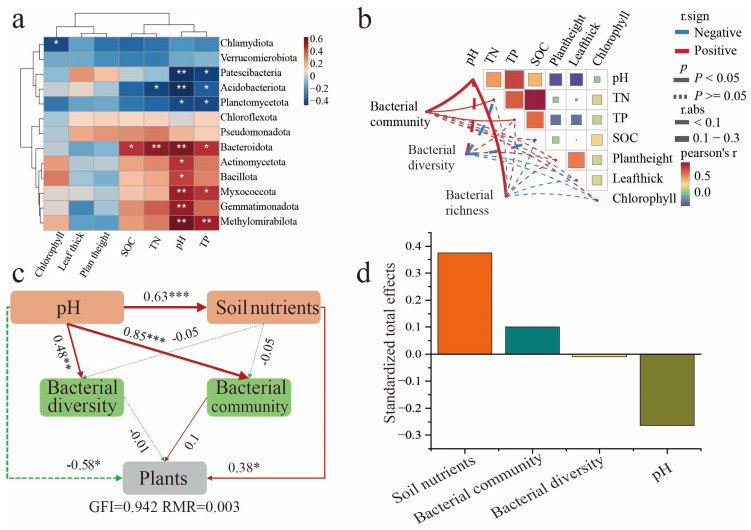
Relationship between soil bacterial community characteristics and soil factors in a *Cymbidium faberi* experiment conducted from July to October 2025. Note: the red solid arrows represent significant positive correlation paths, the green dotted arrows represent negative correlation paths, and the numbers on the arrows indicate the standardized path coefficients. Significance is indicated as * *p* < 0.05, ** *p* < 0.01, and *** *p* < 0.001. TN—total soil nitrogen, TP—total soil phosphorus, SOC—soil organic carbon. (**a**) Representation of correlation heatmap (**b**) Mantel Analysis (**c**) Structural Equation Model (**d**) Analysis of the overall effect value.

The correlation heatmap analysis of the bacterial community phyla of different treated soils with soil environmental factors revealed (Figure 8a) that *Patesibacteria*, *Acidobacterta*, and *Panctomycetota* were significantly negatively correlated with pH and TP, while *Bacteroidota*, *Actinomycetota, Bacillota*, *Myxococcota*, *Gemmatimonadota*, and *Methylomirabilota* were significantly positively correlated with pH (*p* < 0.05). Through Mantel analysis (Figure 8b), it was found that the composition and richness of the bacterial community were significantly positively correlated with pH (*p* < 0.05), while environmental factors had no significant effect on bacterial diversity. This indicates that soil bacterial diversity is influenced by multiple factors together. Further analysis using the structural equation model revealed (Figure 8c,d) that soil nutrients had a significant positive correlation with plant growth, while pH had a significant negative correlation with plant growth (*p* < 0.05). Additionally, total effect analysis showed that soil nutrients and bacterial communities were the main environmental factors affecting plant growth.

## 4. Discussion

### 4.1. Effects of Different Inoculants on the Growth Status of Cymbidium faberi and Soil Nutrients

Applying inoculant agents is an important means to improve the soil quality and enhance the quality of *Cymbidium faberi* [8,20]. This study found that there were significant differences in the effects of different treatments on soil properties and plant growth. Among them, treatments such as KC, HC, and HF all increased soil organic matter, total nitrogen, and total phosphorus. This might be because the microbial fertilizers decomposed the organic matter in the soil and transformed it into inorganic nutrients that plants can absorb, promoting the dissolution and release of insoluble nutrients in the soil and enhancing the soil’s nutrient supply capacity, ultimately improving the nutrient status of *Cymbidium faberi* soil [21]. Under the HKH treatment, plant height and leaf thickness were higher than those in other treatments, while the HC treatment inhibited plant height and chlorophyll content. This might be because the HKH treatment was a ternary compound application, and the three elements worked together to improve the rhizosphere environment, activate nutrients, and promote the synthesis of endogenous hormones in plants, thereby significantly increasing plant height and leaf thickness, showing a strong promoting effect. Conversely, the HC treatment that applied only *Trichoderma harzianum* might have caused short-term stress to the roots due to the high application amount, and it also increased soil pH, affecting the absorption of trace elements, thereby inhibiting plant height and reducing chlorophyll content [22,23]. Correlation analysis showed that pH was significantly positively correlated with SOC, TN, and TP, and significantly negatively correlated with leaf thickness. This might be because the increase in pH promoted the activation of phosphorus and nutrient cycling, enhancing soil fertility. The alkaline environment inhibited the development of plant leaves, resulting in a decrease in leaf thickness [24,25]. TN was significantly positively correlated with SOC and TP, and TP was significantly positively correlated with SOC, indicating a synergistic accumulation effect among soil carbon, nitrogen, and phosphorus. The increase in organic carbon provided a carrier for the fixation of nitrogen and phosphorus, while the increase in nitrogen and phosphorus in turn promoted the stabilization of organic carbon [26]. Plant height and leaf thickness were significantly positively correlated, indicating a synergistic regulatory relationship between plant growth indicators. The increase in leaf thickness provided more space for photosynthesis, thereby promoting plant height growth [27].

### 4.2. The Effects of Different Inoculants on the Characteristics of Soil Bacterial Communities in Cymbidium faberi Soil

Soil bacteria are an important microbial group in soil that play roles such as decomposing organic matter, participating in nutrient cycling and exchange, promoting plant growth, and improving soil productivity. Their diversity and community composition can well represent the health status of the soil [28]. Applying inoculants is currently a common agricultural measure to enhance the composition and relative abundance of beneficial microbial communities in soil and reduce the composition and abundance of pathogenic microorganisms [29]. High-throughput sequencing technology is currently widely used in the study of bacterial diversity and community composition in soil due to its advantages such as high throughput, fast speed, and accurate sequencing results [30]. This study analyzed the soil bacteria in *Cymbidium faberi* soil treated with different inoculants using high-throughput sequencing technology, and after normalization, 12,414 ASVs were obtained, covering a large number of bacterial groups belonging to 35 phyla and 717 genera, indicating that *Cymbidium faberi* soil has abundant bacterial species resources, providing a solid foundation for the stable functioning of soil ecological functions. At the phylum level, the relative abundance of the top 10 dominant phyla accounted for 96.80% of the total abundance, among which *Pseudomonadota* and *Acidobacteriota* were the core dominant groups, which was consistent with the composition characteristics of dominant bacterial phyla in farmland and garden soil in previous studies, confirming that these phyla play a key role in soil material cycling and nutrient transformation [31,32]. Compared with CK, HH treatment increased the relative abundance of *Pseudomonadota* and *Bacteroidota*, while reducing the relative abundance of *Acidobacteriota* and *Verrucomicrobiota*. This may be attributed to the fact that *Pseudomonadota* largely comprise beneficial rhizosphere microbial groups; they can inhibit the proliferation of harmful soil-borne microorganisms—for instance, by competing for ecological niches or secreting antimicrobial substances—thereby enhancing the stability of the rhizosphere micro-ecosystem and the plant’s stress tolerance. *Bacteroidota*, conversely, can accelerate the decomposition of soil organic matter and the mineralization of nutrients, thereby improving the soil’s nutrient-supplying capacity. *Acidobacteriota* and *Verrucomicrobiota* are predominantly adapted to oligotrophic, nutrient-poor environments; consequently, as soil nutrient conditions improve, their competitive advantage diminishes, leading to a decline in their relative abundance [33,34]. At the genus level, HF treatment increased the relative abundance of *Bradyrhizobium*, which may be because this genus can not only form symbiosis with leguminous plants for nitrogen fixation but also promote the development of non-leguminous plant root systems and nutrient absorption. The increase in its abundance helps improve the nutrient supply of *Cymbidium faberi* roots. Conversely, KC treatment significantly reduced the relative abundance of Xiphinematobacter and *Bryobacter*, indicating that Bacillus spores inhibit the growth of these bacteria through competition for nutrients or secretion of antibacterial substances, thereby regulating the root micro-ecological balance [35]. The Venn diagram analysis showed that there were 146 common ASVs among the treatments, indicating that different microbial fertilizer treatments did not change the existence of core bacterial groups in *Cymbidium faberi* soil, while the differences in the number of unique ASVs reflected the specific regulatory effects of the fertilizers on the soil bacterial community. Among them, KC, HC, and HF treatments had more unique ASVs, which was consistent with the results of significantly increasing bacterial diversity and richness by these three treatments, indicating that single-fertilizer treatments are more conducive to promoting the differentiation of soil bacterial communities and the enrichment of specific groups. HK treatment, on the other hand, had the fewest unique ASVs, which may be due to the antagonistic effect between the two reagents, inhibiting the growth and reproduction of some specific bacteria [36,37]. The principal coordinate analysis based on the Bray-Curtis algorithm indicates that the bacterial community structures of different treatments are significantly separated. The HKH treatment shows the greatest difference compared to other treatments and significantly reduces bacterial diversity and richness, suggesting that this treatment causes an imbalance in the soil microenvironment and intensifies bacterial community competition. The HF, KC, and HC treatments cluster together, indicating that the regulation direction of the single microbial agent treatment on the bacterial community structure of *Cymbidium faberi* soil is consistent. This is consistent with the characteristics of the three treatments having a higher number of unique ASVs and significant improvement in diversity, further confirming that a single type of microbial agent is more suitable for the bacterial community of *Cymbidium faberi* soil. These findings directly address our initial hypothesis regarding inoculant type and community responses: contrary to the expectation that combined inoculants would outperform single agents, the data instead show that single-agent treatments were more effective at promoting bacterial diversity and enriching beneficial taxa.

### 4.3. The Impact of Different Inoculant Agents on the Characteristics of the Soil Bacterial Network in Cymbidium faberi

This study, based on the topological analysis of the soil bacterial co-occurrence network, indicates that different inoculant agent treatments have a significant impact on the interaction patterns and network structure of the bacterial community. This impact is not only reflected in the overall change of network complexity, but also profoundly reshapes the types of interactions between species and the topological roles of key species, thereby correlating with the environmental adaptability and stability of the bacterial community [38,39]. This study verified our core hypothesis that inoculant application reshapes soil bacterial network structure, reducing network complexity while improving community stability, with distinct regulatory effects between single and combined inoculant treatments. Overall, different inoculant treatments profoundly altered bacterial interspecific relationships and the topological roles of core species, further driving differences in environmental adaptability and stability of soil bacterial communities. Research shows that the number of nodes, edges, and average weighted degree in the HKH treatment is the highest, indicating that the complexity of the soil bacterial co-occurrence network in this treatment is higher, and the interactions between species are also more complex. This may be because the three additives jointly create a more diverse resource environment, thereby enabling more bacterial groups with different ecological habits to form dense interaction relationships [40]. Further analysis of the proportion of positive and negative correlated edges can provide a deeper understanding of the nature of the interaction relationships. The proportion of positive correlated edges in the HH treatment is the highest, indicating that the cooperative relationship between bacteria occupies a dominant position. This may be because potassium sulfite fulvic acid, as an organic carbon source and biological stimulant, provides a basic energy source for bacteria, while *Trichoderma harzianum* as a beneficial fungus may form mutualistic or partial mutualistic relationships with multiple bacteria, jointly promoting the integration of the community function [41,42]. In the KC and HK treatments, the proportion of negative correlated edges is higher, indicating that the competitive relationship between bacteria occupies a dominant position. This may be because the high-dose Bacillus subtilis, as a dominant bacterium, competes fiercely with the original soil bacteria for nutrients and space, and its secondary metabolites also inhibit the growth of some sensitive groups, resulting in a large number of negatively correlated connections in the network [43,44]. Although this competitive-dominated network structure may be of a relatively high complexity, its stability maintenance mechanism is fundamentally different from the cooperative-dominated network. The Zi-Pi analysis found that in the KC, HF, HK, HH, and HKH treatments, there are more Connectors and Module hubs, indicating that the increase in these two types of nodes jointly constructs a more flexible and resilient community structure. Even if some modules are damaged, the core function of the network can still be maintained through the connection effect of Connectors [45,46,47,48]. In contrast, in the CK, HC, and KH treatments, a large number of nodes are concentrated in the Peripherals area, while Connectors and Module hubs are scarce, reflecting a highly modular network structure with weak connections between modules, and the entire community structure is relatively loose, with poor resistance to external environmental changes [49]. Furthermore, the study found that almost all treatments lacked Network hubs, indicating that in the soil bacterial community, there are very few “core species” that can simultaneously regulate the entire network and all modules. The organization and stability of the community do not rely on a single or a few absolute core species but are maintained through the collaborative and decentralized management of numerous Connectors and Module hubs. This finding provides an important theoretical basis for optimizing the soil microecological function through the precise regulation of microbial community composition.

Correlation analysis revealed that *Patescibacteria*, *Acidobacteriota*, and *Planctomycetota* were significantly negatively correlated with pH and TP, while these microorganisms typically prefer acidic or oligotrophic environments. *Bacteroidota*, *Actinomycetota*, *Bacillota*, *Myxococcota*, *Gemmatimonadota*, and *Methylomirabilota* were significantly positively correlated with pH, indicating that they have a competitive advantage in neutral to alkaline soil conditions. This suggests that pH is a key environmental factor regulating the composition of the soil bacterial community, as it directly affects enzyme activity and the solubility of nutrients, exerting selective pressure on different types of bacteria [50,51]. The Mantel test further confirmed that the overall composition and richness of the bacterial community have a significant positive correlation with pH, while environmental factors did not show a significant impact on bacterial diversity. This indicates that the species composition structure of the community is more sensitive to environmental changes than diversity indicators, and diversity may be regulated by various factors such as interspecies interactions and ecological niche differentiation [52]. Structural equation modeling analyzed the causal relationship between soil environment, bacterial community, and plant growth, revealing that soil nutrients have a significant direct positive effect on plant growth, while pH has a significant direct negative effect on plant growth. Combined with the positive correlation between pH and most beneficial bacterial groups in the correlation analysis, this negative effect may result from an excessively high pH directly limiting the plant’s absorption of certain trace elements, or indirectly altering the rhizosphere microenvironment [53]. Total effect analysis showed that soil nutrients and bacterial communities are important environmental factors affecting plant growth, indicating that in agricultural production, optimizing nutrient management must be accompanied by the attention to regulating the structure of the microbial community.

In summary, this study systematically clarifies the differential effects of single and compound microbial inoculants on soil nutrients, bacterial community diversity, and network stability in *Cymbidium faberi* rhizosphere soil, as well as their subsequent regulation of plant growth. Nevertheless, this study has certain limitations. The experiment was carried out via potted cultivation under controlled laboratory conditions, which cannot fully simulate the complex field soil environment and open ecological conditions of natural *Cymbidium faberi* growth. The short experimental cycle only reflected the short-term response characteristics of soil microbes and plant growth to inoculants, and the long-term dynamic succession law of rhizosphere microbial communities and the sustained effect of inoculants remain unclear. Future research can focus on field verification experiments to explore the stability and persistence of inoculant regulatory effects under natural growth conditions.

## 5. Conclusions

The results of this study indicate that when the three inoculant agents are applied in combination, they significantly promote the plant height and leaf thickness of *Cymbidium faberi*. When a single inoculant agent is applied, it significantly increases the soil pH, TP, SOC and TN contents. Additionally, *Pseudomonadota*, *Acidobacteriota* and *Verrucomicrobiota* are the dominant bacterial phyla among all treatments, and the bacterial communities among inoculant treatments mainly exhibit mutual cooperation. Among them, the proportion of positive correlation edges in the HKH treatment is the highest, and the bacterial interaction is stronger. pH is a significant environmental factor influencing the composition of bacterial communities, and soil nutrients and bacterial communities are the core factors regulating the growth of *Cymbidium faberi*. In summary, this study provides new insights into the characteristics of bacterial communities and nutrient transformation of *Cymbidium faberi* under different inoculant agents.

## Figures and Tables

**Table 1:** Properties of soil and plants in a potted *Cymbidium faberi* experiment conducted from July to October 2025.

Treatments	Soil pH	SOC (g/kg)	TN (g/kg)	TP (mg/kg)	Plant Height (cm)
CK	5.02 ± 0.06 b	23.57 ± 4.05 ab	1.63 ± 0.20 a	306.26 ± 25.43 ab	38.89 ± 3.99 c
HF	6.15 ± 0.09 a	24.15 ± 0.48 ab	1.78 ± 0.02 a	349.73 ± 7.92 a	53.59 ± 4.94 abc
KC	5.72 ± 0.29 ab	20.07 ± 3.70 abc	1.52 ± 0.16 ab	308.75 ± 9.03 ab	47.02 ± 5.47 bc
HC	6.16 ± 0.33 a	17.57 ± 0.59 abc	1.46 ± 0.05 ab	353.77 ± 3.06 a	40.46 ± 2.74 bc
HK	6.22 ± 0.36 a	26.78 ± 6.94 a	1.88 ± 0.34 a	369.80 ± 37.29 a	57.23 ± 5.76 ab
HH	5.43 ± 0.36 ab	19.36 ± 0.52 abc	1.44 ± 0.06 ab	313.66 ± 47.39 ab	49.10 ± 6.10 bc
KH	5.51 ± 0.07 ab	13.77 ± 1.49 bc	1.07 ± 0.08 b	242.21 ± 25.74 b	45.59 ± 7.61 bc
HKH	5.50 ± 0.18 ab	12.49 ± 1.63 c	1.00 ± 0.09 b	281.35 ± 21.74 ab	69.36 ± 3.31 a

Note: CK: control group, no addition of any microbial agents; HF: added mineral sulfosulfuric acid potassium; KC: added Bacillus subtilis; HC: added *Trichoderma harzianum*; HK: added mineral sulfosulfuric acid potassium and Bacillus subtilis; HH: added mineral sulfosulfuric acid potassium and *Trichoderma harzianum*; KH: added Bacillus subtilis and *Trichoderma harzianum*; HKH: added mineral sulfosulfuric acid potassium, Bacillus subtilis, and *Trichoderma harzianum*. Different lowercase letters indicate significant differences after LSD test adjustment among each treatment (*p* < 0.05).

**Table 2:** Basic information of measured indicators in the experiment.

Measured Indicator	Unit	Sample Type	Determination Instrument and Method
Plant height	cm	*Cymbidium faberi* plant	Straight ruler
Leaf thickness	mm	Fresh leaf	Vernier caliper
Relative chlorophyll content	SPAD	Fresh leaf	SPAD-502 Plus chlorophyll analyzer (Konica Minolta, Osaka, Japan)
Soil pH	—	Air-dried rhizosphere soil	pH meter (soil–water ratio 2.5:1)
Soil organic matter	g/kg	Air-dried rhizosphere soil	Potassium dichromate titration method
Soil total nitrogen	g/kg	Air-dried rhizosphere soil	Automatic Kjeldahl nitrogen analyzer (Beijing BeifenRuili Analytical Instrument Co., Ltd., Beijing, China)
Soil total phosphorus	mg/kg	Air-dried rhizosphere soil	HClO_4_-H_2_SO_4_ digestion method
Soil microbial DNA	—	Fresh rhizosphere soil (−80 °C)	FastDNA^®^ SPIN Kit, NanoDrop 2000 (Thermo Fisher Scientific, Wilmington, MA, USA)

**Table 3:** Overview and application scope of statistical analysis methods.

Statistical Method	Research Purpose	Interpretation and Application Scope
Shapiro–Wilk, Bartlett and Levene test	Test normality and homogeneity of variance	Verify the preconditions for one-way ANOVA
One-way ANOVA combined with LSD multiple comparison	Compare differences in plant and soil properties	Significance difference of each index among all treatments at *p* < 0.05
Principal component analysis (PCA)	Analyze overall differentiation of bacterial community structure	Community composition similarity and variation pattern across treatments
ANOSIM analysis	Test statistical difference of bacterial community composition	Judge whether community structure differs significantly among treatments
LEfSe analysis	Screen differential biomarker bacterial taxa	Identify genus-level bacteria with significant abundance differences
Bacterial molecular ecological network	Explore interspecific interaction of bacterial genera	Reveal co-occurrence and antagonistic relationships of microbiota
PLS-PM path analysis	Clarify complex influencing pathways	Reveal direct and indirect effects of microbial agents on soil bacterial community

## Data Availability

The sequence data associated with this project have been deposited in the NCBI database under accession number PRJNA1454210.
